# The breadth of HIV-1 neutralizing antibodies depends on the conservation of key sites in their epitopes

**DOI:** 10.1371/journal.pcbi.1007056

**Published:** 2019-06-06

**Authors:** Hongjun Bai, Yifan Li, Nelson L. Michael, Merlin L. Robb, Morgane Rolland

**Affiliations:** 1 U.S. Military HIV Research Program, Walter Reed Army Institute of Research, Silver Spring, MD, United States of America; 2 Henry M. Jackson Foundation for the Advancement of Military Medicine, Bethesda, MD, United States of America; ETH Zurich, SWITZERLAND

## Abstract

Developing HIV-1 vaccines that trigger broadly neutralizing antibodies (bnAbs) is a priority as bnAbs are considered key to elicitation of a protective immune response. To investigate whether the breadth of a neutralizing antibody (nAb) depended on the conservation of its epitope among circulating viruses, we examined Antibody:Envelope (Ab:Env) interactions and worldwide Env diversity. We found that sites corresponding to bnAb epitopes were as variable as other accessible, non-hypervariable Env sites (p = 0.50, Mann-Whitney U-test) with no significant relationship between epitope conservation and neutralization breadth (Spearman’s ρ = -0.44, adjusted p = 0.079). However, when accounting for key sites in the Ab:Env interaction, we showed that the broadest bnAbs targeted more conserved epitopes (Spearman’s ρ = -0.70, adjusted p = 5.0e-5). Neutralization breadth did not stem from the overall conservation of Ab epitopes but depended instead on the conservation of key sites of the Ab:Env interaction, revealing a mechanistic basis for neutralization breadth that could be exploited for vaccine design.

## Author summary

So far, no HIV-1 vaccine has elicited broadly neutralizing antibodies (bnAbs) in humans. HIV-1, one of the most rapidly evolving pathogens, is remarkable for its high variability across individuals and adaptability within hosts. We tested the relationship between HIV-1 diversity and neutralization breadth. While bnAbs did not specifically target more conserved regions of HIV-1 Env, we found that the broadest bnAbs relied forcibly more on structural interactions at key sites of the Ab:Env interaction than other Abs. Understanding mechanisms underlying neutralization breadth provides guidelines to design more efficacious vaccines and antibody-based therapeutics.

## Introduction

There is an urgent need for a vaccine against HIV-1. Since HIV-1 shows remarkable diversity, it is assumed that a vaccine should elicit bnAbs to block the most extensive array of HIV-1 strains[1–4]. Neutralizing Abs develop over the course of HIV-1 infection and there is a continuum in the extent of neutralization breadth developed across individuals, with typically half of a cohort being able to neutralize about half of a virus panel [5]. A number of studies have focused on the fraction of individuals who can develop bnAbs that can neutralize a majority of the viruses in a panel[6–9]. These bnAbs arise after Ab lineages have matured typically over multiple years[5, 10–13]. Highlighting the many paths that can lead to bnAb development, bnAbs have been isolated from individuals with different HIV-1 subtypes and presenting different clinical disease progression profiles. For example, VRC01 was isolated from patient 45, an African-American male who had been infected with HIV-1 subtype B for 11 years at the time of Ab isolation; he was considered a long term non-progressor as his viremia was maintained around 10,000 copies/ml [14]. In contrast, the bnAb CH103 was isolated from patient CH505, a male from Malawi who had been infected with HIV-1 subtype C for 2.5 years when Ab were isolated; patient CH505 was followed for six years and maintained a high median viral load of 173,667 over that time [15]. BnAbs recognize exposed regions of the Env trimer and tend to target five sites: the V1V2-glycan site (e.g. PG9), the V3-glycan site (e.g. PGT128), the CD4 binding site (e.g. VRC01), the gp120-gp41 interface (e.g. 8ANC195, 35O22) and the membrane proximal region of Env-gp41 (e.g. 10E8) [16].

It is generally believed that bnAbs target conserved epitopes on HIV-1 Env trimers [17–20]. Yet, no study has systematically quantified the relationship between the neutralization breadth of bnAbs and the conservation of their respective epitopes on Env. Here we analyzed publicly-available Ab:Env complex structures and characterized how the neutralization breadth of an Ab was influenced by the conservation of its epitope. We describe how neutralization breadth was positively associated with Env epitope conservation only when the epitope conservation was defined by taking into account the strength of the Ab:epitope interaction, i.e. specifically weighting structurally important sites, and not simply sequence conservation.

## Results

### Broadly neutralizing antibodies did not specifically target more conserved regions of HIV-1 Env

**Fig 1:**
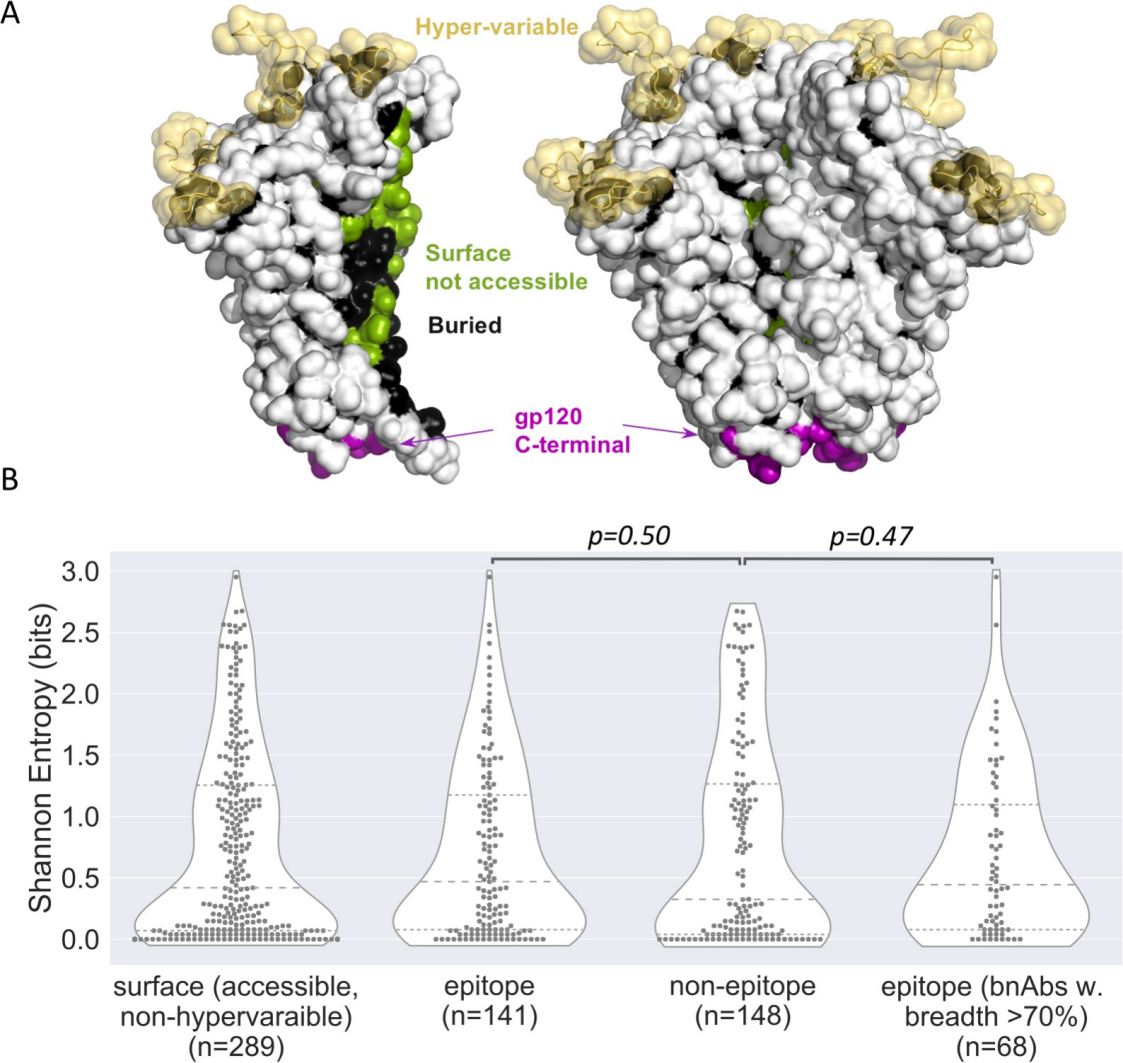
Epitopes of broadly neutralizing antibodies were as variable as other accessible Env sites. (A) Accessible, non-hypervariable surface sites shown on the structure as white. Hypervariable loops and Ab-inaccessible (surface not-accessible, buried, and gp120 C-terminal) sites are excluded from the comparison. The prefusion trimer structure 5FYJ [22]) were used for the accessibility estimation. (B) Shannon entropy of accessible, non-hypervariable Env sites. These sites are further divided to epitope sites and non-epitope sites.

**Fig 2:**
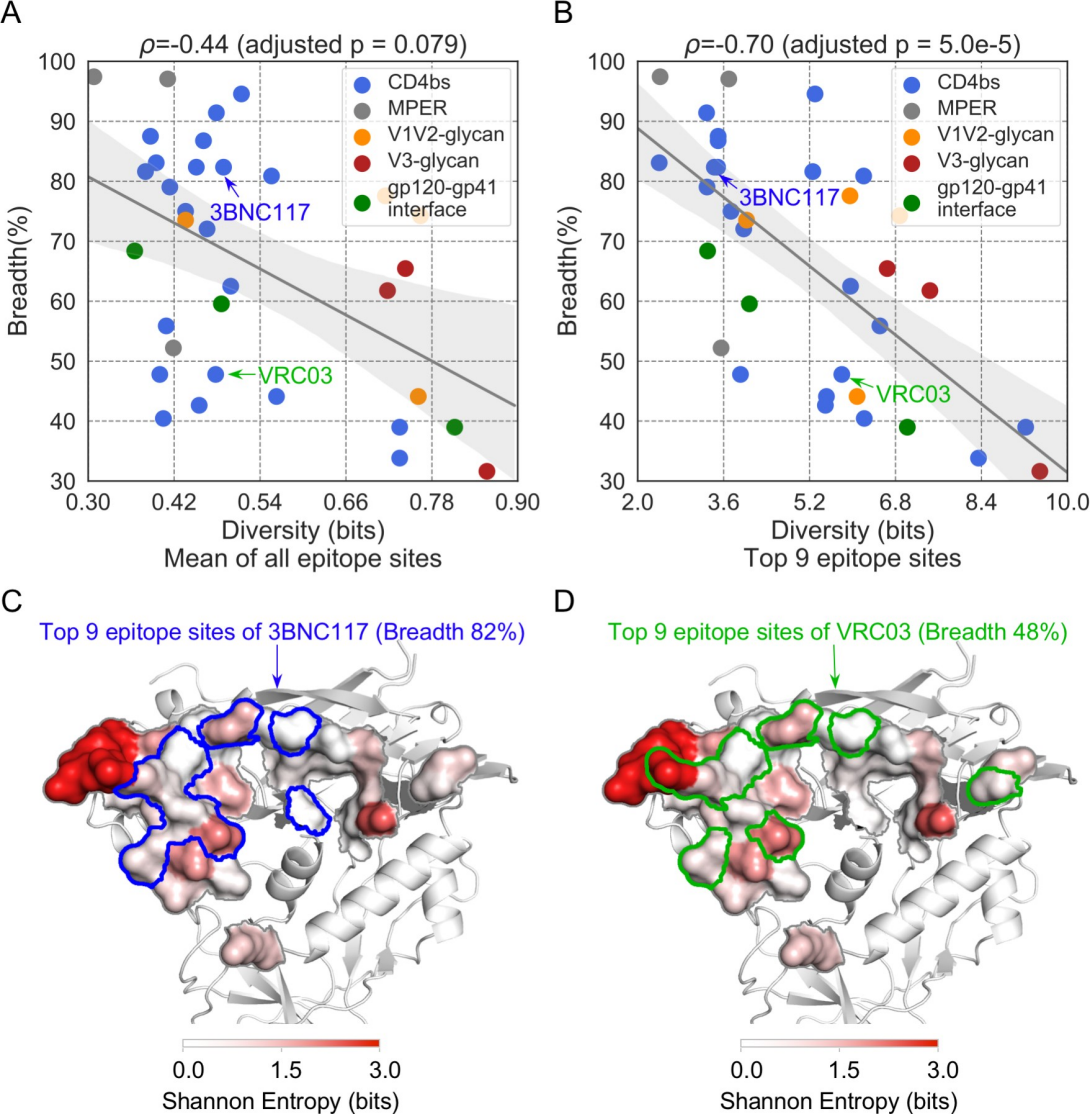
Conservation at key epitope sites was associated with neutralization breadth. Relationship between the neutralization breadth of Abs and (A) the mean diversity of all epitope sites (i.e., normalized for epitopes ranging from 8 to 36 sites) and (B) the top-nine epitope sites (ranked by the number of neighboring Ab residues). The 95% confidence interval of the linear regression line was determined by 1000 bootstrap replicates. The Abs 3BNC117 and VRC03 share similar epitopes but the epitope diversity based on their top nine sites (ranked by the number of neighboring Ab residues) was lower for 3BNC117 than that for VRC03; 3BNC117 was more focused on conserved epitope sites (C) than VRC03 (D) and its neutralization breadth was 34% higher than that of VRC03.

**Fig 3:**
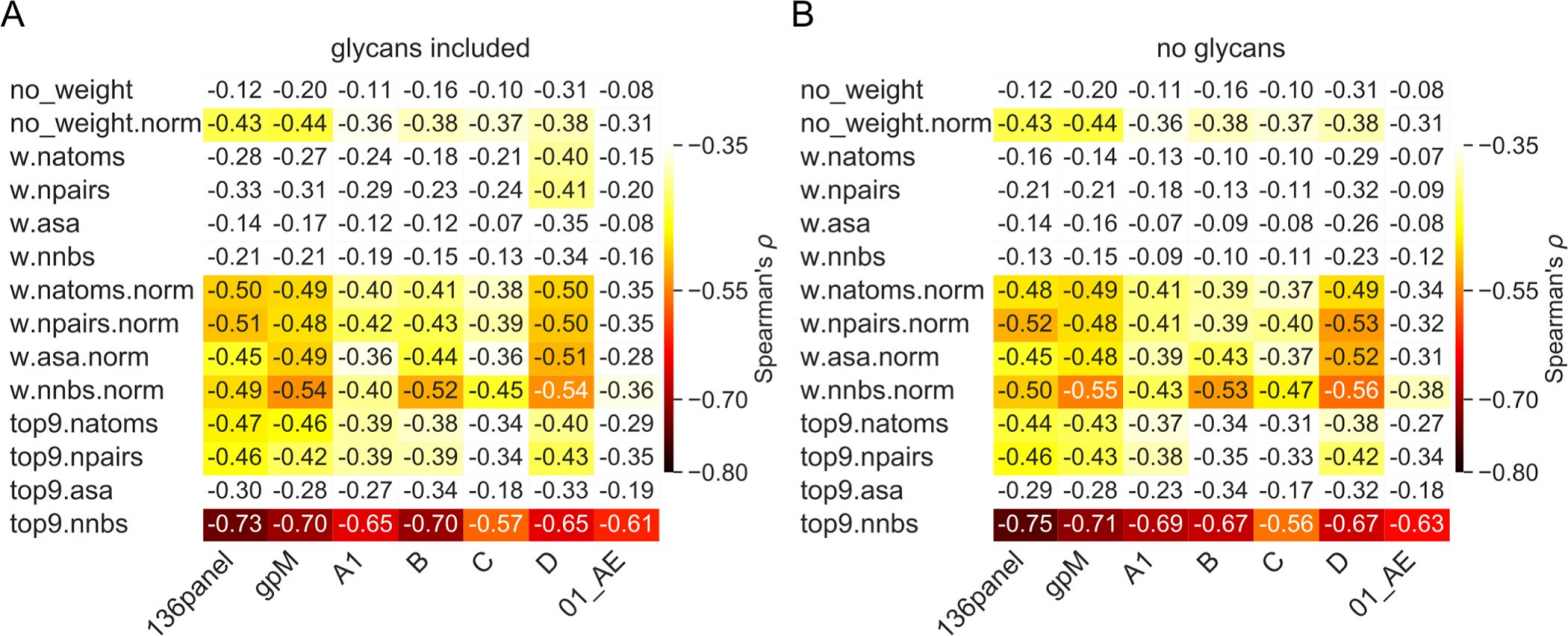
Relationship between neutralization breadth and Env epitope diversity. Epitope diversity measures were calculated using different Env dataset (columns) and different weighting schemes (rows). Spearman’s ρ coefficients corresponding to the relationship between neutralization breadth and Env epitope diversity are presented with (A) and without glycans (B) included in the calculations and color-coded for ρ values that showed a p-value < 0.05. The Env datasets tested are the panel of 136 viruses experimentally-assayed for neutralization (136panel), the global distribution of HIV-1 (gp M), and five subtype-specific alignments (A1, B, C, D and CRF01_AE). In rows, epitope sites were not weighted (no_weight), or weighted by the number of Ab atoms contacted (w.natom), the number of atom pairs in contact between the epitope and the Ab (w.npairs), the reduced accessible surface area after Ab binding (w.asa) and the number of neighboring Ab residues in the Ab:Env complex (w.nnbs). If only the top nine epitope sites were selected for diversity estimations, a prefix ‘top9’ was applied. Epitope diversity values normalized for the size of the epitope are indicated with the suffix ‘norm’.

To characterize the diversity of HIV-1 circulating strains, we created an HIV-1 group M alignment of 239 Env sequences that reflected the global representation of HIV-1 subtypes (gp M), as well as specific datasets for subtype A1 (n = 203), B (n = 1035), C (n = 1184), D (n = 116) and CRF01_AE (n = 577). We analyzed 34 Abs for which neutralization breadth had been measured using a panel of 136 viruses and ranged between 31 and 97% [21] and for which Ab:Env complex structures were available (S1 Table, S1 Fig). For these 34 Abs, the epitope consisted of 8 to 36 sites. We looked at the diversity among group M sequences at each accessible, non-hypervariable site on the surface of Env and found no difference between sites that belonged to Ab epitopes (n = 31 Abs, epitopes of 3 MPER antibodies were excluded as they are partially/totally missing from the Env structure 5FYJ, which was used to define surface sites) and sites that were outside of epitopes: median Shannon entropy = 0.47 vs. 0.32 bits, respectively (p-value = 0.50, Mann-Whitney U-test) (Fig 1). This result was confirmed when the analysis was restricted to the 15 antibodies that showed over 70% breadth: median Shannon entropy = 0.44 vs. 0.32 bits, respectively (p-value = 0.47, Mann-Whitney U-test) (Fig 1B). Thus, bnAbs targeted Env sites that were as variable as other accessible Env sites. To define the epitope diversity, we summed the Shannon entropy of all the epitope sites and adjusted with the mutual entropy of neighbor pairs of sites. This corresponds to the diversity of the whole epitope patch, which we then normalized based on the size of the epitope. There was no relationship between the epitope diversity (or conservation) and the breadth of neutralization of HIV-1 strains by nAbs if we considered the epitope diversity of the whole epitope (Spearman’s ρ = -0.20, adjusted p-value = 0.70) or when the epitope diversity was normalized based on the size of the epitope (Spearman’s ρ = -0.44, adjusted p-value = 0.079; Fig 2A) when using the set of sequences representative of the global HIV-1 distribution. Results were similar when we tested the sequence sets corresponding to different subtypes/CRF: Spearman correlation coefficient ρ ranged between -0.31 and -0.43 with adjusted p-values > 0.078, showing that the finding was not dependent on the dataset tested (group M versus subtype-specific alignments) (Fig 3).

### Structurally-weighted conservation of key epitope sites associated with increased neutralization breadth

We modified our definition of epitope diversity to integrate structural factors beyond sequence conservation that may be critical to efficient Ab neutralization. These epitope diversity measures weighted each site in the Env epitope by i) the number of Ab atom contacts for each Env epitope residue, ii) the number of atom pairs in contact between the epitope and the Ab, iii) the number of neighboring Ab residues for a given epitope residue in the Ab:Env complex and iv) the reduced accessible surface area after Ab binding. These definitions were also normalized for the size of the epitope (8–36 sites). The results reported below correspond to the nine epitope sites that had the highest number of neighboring Ab residues (we focused on nine sites as it showed the best correlation when comparing between seven and eleven key sites, S2 Fig). Fig 3 shows the effect of weighting by the features described above, we found a negative relationship between epitope diversity and neutralization breadth: the more conserved the structurally weighted Env epitope, the greater the neutralization breadth. If we consider the alignment of 239 representative group M sequences, the Spearman correlation coefficient ρ was -0.70 (adjusted p-value = 5.0e-5) (Fig 2B, S2 Table). The negative relationship between the Env epitope diversity and neutralization breadth was replicated when restricting the analysis to specific subtypes/CRF: considering the top nine target sites, the Spearman correlation coefficient ρ ranged between -0.57 and -0.73 with adjusted p-values ≤ 6.1e-3. We note that the relationship was similar when we considered only the dataset corresponding to the subtype matching the subtype of the infected individual from whom the Ab was obtained (Spearman’s ρ = -0.70, adjusted p-value = 1.7e-3; S3 Fig and S4 Fig). Since most of the 34 Abs targeted the CD4 binding site (CD4bs) (n = 21), we analyzed data separately for these Abs. We found that the relationship between epitope diversity and neutralization breadth was largely driven by CD4bs Abs (Spearman’s ρ ranged between -0.61 and -0.73 with adjusted p-values ≤ 3.1e-3 when analyzing the different sequence sets, S3 Table). However, for the 13 other Abs, the relationship was not improved by the structural-weighting (Spearman’s ρ ranged between -0.39 and -0.66, 0.18 ≤ adjusted p-values ≤ 1.00, S3 Table; the lack of significance may be due to the small sample size).

The fact that epitope conservation failed to strictly derive from sequence conservation but corresponded to a structurally-weighted conservation measure is illustrated by the comparison of the Env epitopes of 3BNC117 and VRC03, two Abs that target the CD4 binding site. The bnAb 3BNC117 neutralizes 82% of HIV-1 strains while VRC03 neutralizes 48% of HIV-1 strains. Because the Env epitopes of 3BNC117 and VRC03 are very similar, the unweighted measure of diversity gave similar diversity values. However, when we considered only the structurally key epitope sites for Ab binding, 3BNC117 engaged conserved sites while VRC03 had many atom contacts with more variable sites such as amino acids 460 and 461 in Env-V5. Hence, the VRC03 epitope had a higher diversity value for its top nine sites than the 3BNC117 epitope and was associated with a more limited breadth of neutralization, highlighting that the mode of interaction between the Ab and epitope and not just the location of the epitope was associated with increased neutralization coverage (Fig 2).

**Fig 4:**
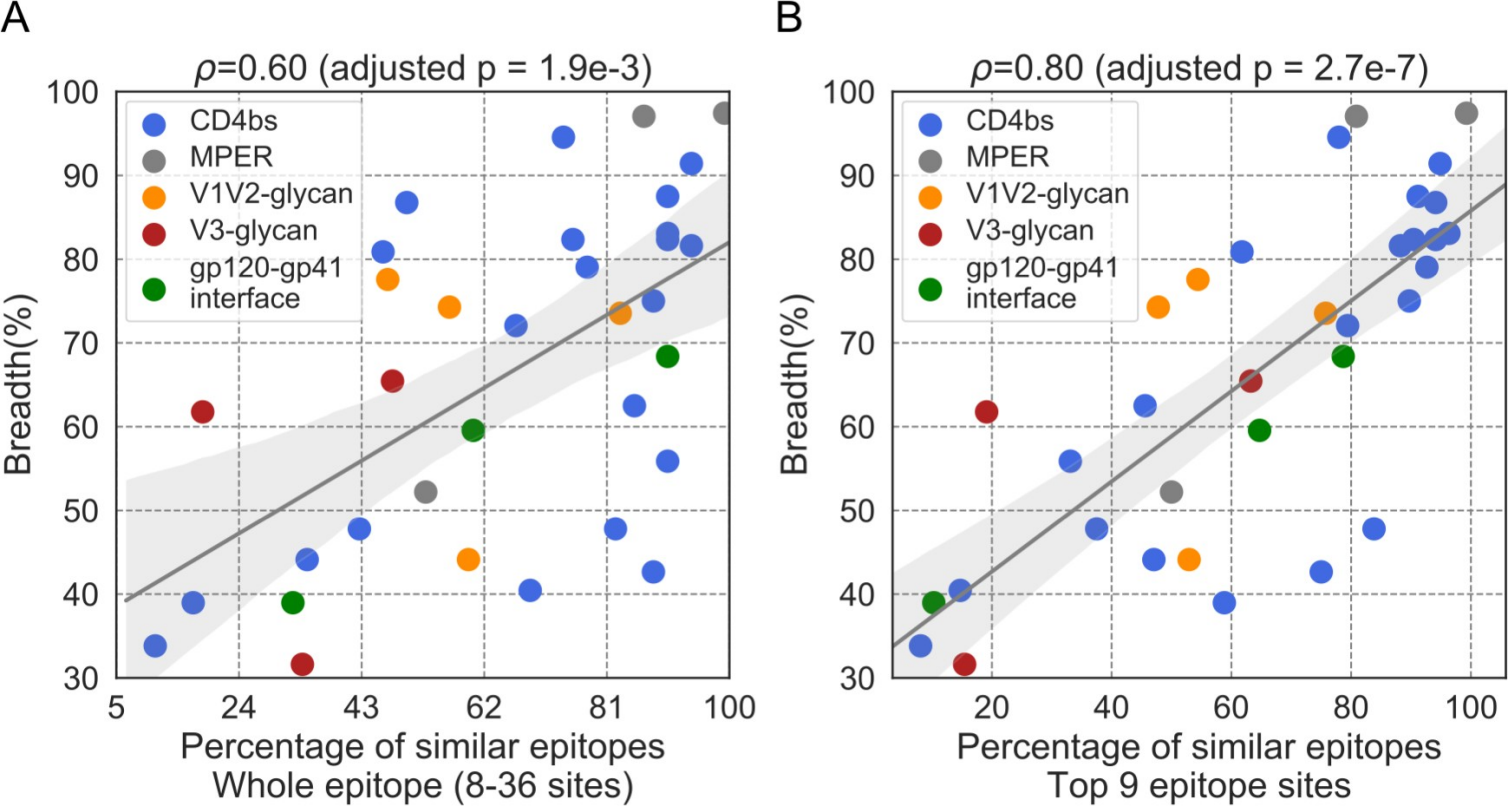
Relationship between neutralization breadth and the epitope similarity to susceptible strains. The epitope similarity of a given antibody was calculated by comparison to the three most susceptible strains to this antibody in a panel of 136 viruses; values are reported for the whole epitope (A) or only for the top 9 epitope sites (B). The 95% confidence interval of the linear regression line was determined by 1000 bootstrap replicates.

We obtained similar results when we used as a measure of epitope conservation the similarity of a given epitope to its counterpart in the three strains experimentally-defined as the most susceptible to neutralization. The three most susceptible strains were selected using the data from the panel of 136 viruses assayed by Doria-Rose and colleagues [21] (up to five strains were tested before choosing a combination of three strains, S5 Fig). For each Ab, we calculated the fraction of viruses with epitopes matching the epitope in the three most susceptible strains. When we considered whole epitopes, the nAbs that showed a higher fraction of epitopes similar to the three most susceptible strains were associated with increased neutralization breadth (Spearman’s ρ = 0.60, adjusted p = 1.9e-3). As seen above, this relationship was stronger when we focused on the top nine epitope sites (ranked by the number of neighboring Ab residues) in the Ab:Env interaction: Spearman’s ρ = 0.80, adjusted p = 2.7e-7 (Fig 4). Similar to what we noted above, the Spearman’s correlation coefficient was stronger for Abs that targeted the CD4bs (Spearman’s ρ = 0.78, adjusted p = 5.2e-4, S4 Table) than for Abs that targeted other epitopes (Spearman’s ρ = 0.73, adjusted p = 0.070, S4 Table).

### Impact of Env glycans

One shortcoming of this study is that Env accessibility was measured using Env structures with glycans removed, while we know that HIV-1 Env trimers are covered by a glycan shield of ∼90 N-linked oligosaccharides constituting about half of the Env mass—a key factor in HIV-1 evading humoral immunity[22–24]. We weighted the epitope diversity calculations (described above) for the presence of glycans at specific epitope sites, yet this modification did not reveal any significant difference in the relationship between epitope diversity and neutralization (Fig 4). We compared the 9-, 7- and 5-mannose models sampled in molecular dynamics simulations [25] and found that the median Ab accessibility was diminished by about three-fold when glycans were integrated: 13.6 Å in the 9-mannose model vs. 4.6 Å in the absence of glycans. The 7- and 5- mannose models provided better Ab accessibility than the 9-mannose model (median depth of 11.1 Å, 12.2 Å and 13.6 Å for 5-, 7 and 9-mannose Env models, respectively), indicating that engineering the glycan shield to have only 5 or 7 mannose residues at each glycan site may improve Ab accessibility. This is consistent with the finding that restricting the glycan site to be 5 mannose greatly increased its susceptibility to an array of bnAbs[26].

## Discussion

In summary, we systematically analyzed the interaction between nAbs and their corresponding Env epitopes to identify the mechanistic basis of HIV-1 neutralization breadth. Surprisingly, although it is widely accepted that HIV-1 bnAbs target conserved segment of HIV-1 Env, we demonstrated that bnAbs targeted Env sites that were no more conserved than other accessible, non-hypervariable Env sites and that the breadth of a nAb was not significantly related to the conservation of its epitope among circulating viruses if we used a standard measure of epitope conservation. It is only when HIV-1 conservation was measured by accounting for the structural strength of the Ab:Env interaction that we found a positive relationship between sequence conservation and neutralization breadth. We note, however, that certain factors complicated our analysis. For example, it is difficult to account for the influence of the glycan shield or for the missingness of some structural information such as for MPER antibodies where only a fragment of Env is complexed with the antibody. Future studies will also be needed to evaluate whether non-neutralizing antibodies differ from bnAbs in their mode of interaction with Env.

Our finding has implications for vaccine development. We showed that epitopes were not more conserved than any other non-hypervariable sites at the surface of the prefusion-closed Env trimer, yet the broadest bnAbs showed key interactions at very conserved sites. Our study indicates that targeting conserved epitopes is necessary but not sufficient to promote breadth and that an antibody’s interactions with key conserved sites are primordial to achieve neutralization breadth. This would suggest that targeting conserved epitopes may not be sufficient if there is no further Ab maturation to focus on key epitope sites. Hence, this work suggests Ab breadth could be improved by designing epitope patches on immunogens to favor breadth-promoting Ab interactions. In addition, this knowledge can directly be used to characterize breakthrough and rebound viruses following bnAb-based interventions, providing a novel blueprint to prospectively interpret results of clinical trials that use bnAbs as a therapeutic agent.

## Materials and methods

### Datasets

#### Env sequence alignments

HIV-1 sequences were retrieved from the Los Alamos HIV Database (http://www.hiv.lanl.gov/) on 06/03/2018 for subtype A1, B, C, D, CRF01_AE and the 2017 filtered web alignment. The datasets were curated to retain only independent sequences (one sequence per individual) with a correct open reading frame (ORF); we removed sequences i) with no time stamp or subject identifier, ii) with frame shifts, stop codon or ambiguous residues, and iii) with evidence of hypermutation or recombination. The group M alignment was down-selected from the 2017 filtered web alignment to reflect the proportion of HIV-1 circulating sequences as described in Hemelaar and colleagues [27]: 12% subtype A, 11% subtype B, 48% subtype C, 2% subtype D, 5% subtype G, 5% CRF01_AE, 8% CRF02_AG and 9% of other subtypes and CRF. Sequences were aligned using MAFFT [28] and viewed with AliView [29].

#### Ab:Env complexes

Doria-Rose and colleagues tested 46 HIV-1 Abs against a panel of 136 virus strains to determine their neutralization breadth [21]. Ab:Env complexes were available from the RCSB PDB (as of November 21, 2017) [30] for 34 Abs, which we used for this study (S1 Table). Protein structures were viewed, analyzed and rendered using PyMol (https://pymol.org/2/).

### Epitope definition

An Ab epitope was defined based on the Ab:Env complex structure as the Env sites with heavy atoms (called atoms below and in the main text) that were located within 4 Å of the Ab. Weights were assigned to specific Env sites in different ways, using: i) the number of Ab atoms contacted (w.natom), ii) the number of atom pairs in contact between the epitope and the Ab (w.npairs), iii) the number of neighboring Ab residues in the Delaunay tetrahedralization of C_β_ (C_α_ of Gly) atoms of the Ab:Env complex (w.nnbs), and iv) the reduced accessible surface area after Ab binding (w.asa) [31]. The Delaunay tetrahedralization was obtained using Quickhull [32] and edges longer than 8.5 Å were removed. If multiple complex structures were available for an Ab, the mean weight from the different complex structures was used as the final weight of the epitope. To avoid overweighting, the weight was capped at the 98^th^ percentile of all sites; any site with a weight above the 98^th^ percentile was set to the 98^th^ percentile. The weight of N-linked glycans was scaled such that its 98^th^ percentile was equal to the 98^th^ percentile of the amino acids before adding to the corresponding asparagine.

### Epitope diversity

We defined the epitope diversity as the Shannon entropy [33] of the epitope:

$$H=\sum_{i}H_{i}-\sum_{i,j}I_{i,j}$$ (1)

$$H_{i}=-\sum_{k}p(k)\log_{2}p(k)$$ (2)

$$I_{i,j}=\sum_{m,n}p_{i,j}(m,n)\log_{2}\frac{p_{i,j}(m,n)}{p_{i}(m)∙p_{j}(n)}$$ (3)

where *H*_*i*_ is the Shannon entropy of epitope site *i*; *I*_*i*,*j*_ is the mutual entropy between a pair of neighbor sites *(i*,*j); p(k)* is the fraction of amino acid *k* on a site; *p*_*i*,*j*_ (*m*,*n*) is the fraction of amino acid combinations *(m*,*n)* on a pair of neighbor sites *(i*,*j)* (*m* on site *i* and *n* on site *j*). The summation is over all epitope sites in the first term and over all neighbor sites in the second term of Eq 1. In Eq 2, the summation is over all amino acids. In Eq 3, the summation is over all amino acid combinations on neighbor sites *i* and *j*. All neighbor pairs were identified by Delaunay tetrahedralization of C_β_ (C_α_ of Gly) atoms in the ab:Env complex structure. An example based on a toy epitope is provided in the supplementary material to illustrate how the diversity is estimated.

To account for the contribution of specific sites in the Ab:Env complex, we used:

$$H=\sum_{i}w_{i}H_{i}-\sum_{i,j}w_{i,j}I_{i,j}$$ (4)

$$w_{i,j}=(w_{i}H_{i}+w_{j}H_{j})/(H_{i}+H_{j})$$ (5)

where *w*_*i*_, *w*_*j*_ and *w*_*i*,*j*_ are the weights assigned to epitope sites *i*, epitope site *j*, and a pair of neighbor sites *(i*,*j)*, respectively. The summation is over all epitope sites in the first term and over all neighbor pairs in the second term of Eq 4. When the epitope diversity is normalized, the weight of each site is adjusted as $w_{i}^{\prime }=w_{i}/\sum_{i}w_{i}$, in which the summation is over all epitope sites.

Based on the chemical similarity between certain amino acids, we grouped amino acids D and E as ‘a’, R and K as ‘b’, N and Q as ‘n’, L and M as ‘l’, V and I as ‘i', and F and Y as ‘f’ before estimating the Shannon entropy. If N was a potential N-linked glycosylation site, it was flagged as ‘g’.

### Epitope similarity

The epitope similarity between a sequence X and a reference sequence R was defined as:

S(R,X)=−[M(R,R)−M(R,X)] (6)

$$M(R,X)=[\sum_{i}w_{i}∙Sim(R_{i},X_{i})]/\sum_{i}w_{i}$$ (7)

where *M(R*, *X)* is the match score between R and X, *w*_*i*_ is the weight assigned to epitope site *i*, and *Sim(R*_*i*_, *X*_*i*_*)* is from either the BLOSUM62 [34] or the VTML200 [35] matrix, which describes the similarity between *R*_*i*_ (amino acid on site *i* of R) and *X*_*i*_ (amino acid on site j of X). The minus sign on the right side of Eq (6) converted a distance to the similarity.

To avoid relying on a single strain as the reference, we selected three references strains (n one to five strains with lowest IC50s from 136 strains were tested). The highest epitope similarity to the three references were used as the epitope similarities to the susceptible strains (*S*(*R*,*X*) = max(*S*(*R*1,*X*),*S*(*R*2,*X*),*S*(*R*3,*X*))). Then, we predicted the breadth of an antibody as the fraction of strains with an epitope similarity below a similarity threshold (TH). The number of resistant viruses predicted by the epitope similarity should be same as the number of resistant viruses determined in the neutralization assays. Thus, we set the threshold such that ∑*N*_*IC*50≥25μg/ml_ = ∑*N*_*Sim*<*TH*_, in which the summation is over all 34 antibodies. Specifically, given the 136 viruses tested against 34 antibodies, there were 34×136 = 4,624 epitope similarities. The neutralization assays identified 1,561 = 33.8% of virus-Ab combinations as resistant (with IC50 ≥ 25 μg/ml). Thus, we set the similarity threshold as the 1561^th^ element after all the 4,624 epitope similarities were sorted ascendingly.

### Ab accessibility

#### Selection of representative molecular dynamics conformations

Molecular dynamics (MD) simulation trajectories from glycosylated trimers were provided by Cinque Soto and Peter Kwong [25]. The root mean square difference (RMSD) was calculated between all sampled conformations in the MD simulation trajectories using the GROMACS package [36], with C_α_ atoms selected for the superposition of conformations. Two conformations with RMSD below 3.0 Å were considered as neighbors. We selected the conformations that had the maximum number of neighbors and removed neighbor conformations in an iterative process until all conformations were processed. The remaining conformations (439, 360 and 295 conformations for the 9-, 7- and 5-mannose models, respectively) were extracted using MDAnalysis[37, 38].

#### Ab accessibility

The Ab accessibility was measured as the distance between an Env surface site and an Ab probe with a radius of 15 Å rolled over the Env structure; more specifically, it corresponded to the mean distance of heavy side chain atoms (C_α_ included) to the surface of the Ab probe (r = 15 Å) as mapped on the pre-fusion Env-trimer structure (PDB code: 5FYJ [25] in the absence of glycosylation and on the conformations selected from the 5-, 7-, and 9-mannose models as described above. The accessible surface to the Ab probe was approximated by the Shrake-Rupley algorithm [39]. In brief, evenly distributed points (n = 256) were placed on spheres that were centered at atoms in the protein. The radius of those spheres was the radius of the atoms plus the radius of the Ab probe. After filtering out points on a sphere that were within neighbor spheres, the remaining points were clustered by a density-based clustering method [40] to define the accessible surface (i.e., the largest cluster). Note that the Ab probe radius (15 Å) is about the size of the Fv domain of the heavy chain of an Ab. The accessible surface area was calculated by the same procedure without the clustering, with a probe of 1.5 Å and denser sampling points (n = 2048). The surface sites are identified by the side chain relative accessible surface area (ASA), which is defined as the ratio of side chain (Cα included) ASA to the maximum possible ASA (scASA/MaxASA). If a site with scASA/MaxASA > 0.08, it is a surface site. A surface site with a depth smaller than 15 Å is considered as accessible.

### Statistical analysis

Data analysis, visualization and statistical testing were performed in the Python environment[41–47]. Statistical details of analyses can be found in the main text and figure captions where applicable; significance was established at p < 0.05. A link to the data archive and code to reproduce the analysis is provided below.

## Supporting information

S1 Table Thirty-four Abs included in the study. The targets of the Abs on Env, their neutralization breadth as measured by Doria-Rose and colleagues [21] and the names of the Ab:Env complexes in the PDB archive are listed.
(DOCX)

S2 Table Relationship between neutralization breadth and different Env epitope diversities. Spearman’s ρ and adjusted p-values (in parentheses) are presented. Holm–Bonferroni method was used for multiple test adjustment within each sequence dataset.
(DOCX)

S3 Table Relationship between neutralization breadth and different Env epitope diversities. Spearman’s ρ and adjusted p-values (in parentheses) are presented. Holm–Bonferroni method was used for multiple test adjustment within each sequence dataset. CD4bs and non-CD4bs antibodies are shown separately.
(DOCX)

S4 Table Relationship between neutralization breadth and percentage of similar epitope to susceptible strains. The epitope similarity is estimated based on sequences in the 136 panel. Spearman’s ρ and adjusted p-values (in parentheses) are presented. Holm–Bonferroni method was used for multiple test adjustment.
(DOCX)

S1 Fig Location of typical epitope sites on the structure and sequence. (A) Representative bnAb epitopes from our dataset are represented on the structure 5FYJ. (B) Epitope sites on the Env surface sites. The name and breadth of the 34 Abs analyzed are figured as row headers. Histograms correspond to the number of neighbor antibody residues, with the top nine sites colored by epitope categories. Buried sites are included only if they are among the top nine sites. 5FYJ was used for the structure rendering and the surface detection.
(TIFF)

S2 Fig Comparison of the relationship between antibody epitope diversity and neutralization breadth when testing seven to eleven representative epitope sites (top sites). Epitope diversity values were estimated using different Env dataset (columns) and different sets of top sites (rows). Spearman’s ρ coefficients corresponding to the relationship between neutralization breadth and Env epitope diversity are presented with (A) and without glycans (B). Each cell is color-coded for ρ values with a p-value < 0.05. Epitope diversity values of the top nine sites as ranked by the number of neighbor antibody residues showed the highest correlation with the Ab neutralization breadth.
(TIFF)

S3 Fig Relationship between neutralization breadth and the epitope diversity estimated from the HIV-1 subtype matching the subtype of the infected individual (host subtype). (A), relationship between neutralization breadth and the epitope diversity that is estimated from the host subtype sequences using different weighting schemes (rows). Spearman’s ρ and adjusted p-values (Holm–Bonferroni method, in parenthesis) are presented. (B), the neutralization breadth versus epitope diversity of top nine epitope sites estimated from the host subtype sequences. The 95% confidence interval of the linear regression line was determined by 1000 bootstrap replicates.
(TIFF)

S4 Fig Epitope diversity of the host subtype (the HIV-1 subtype of the infected individual from whom the antibody was isolated) compared to other subtypes. (A), the diversity of the top nine epitope sites (ranked by number of neighbor antibody residues) is shown as a heatmap with antibodies in rows and Env alignments in columns. The host subtype is indicated by blue rectangles in the heatmap. (B), the three panels on the right compare the epitope diversity of the host subtype against other subtypes (with corresponding host subtypes excluded).
(TIFF)

S5 Fig Relationship between neutralization breadth and the epitope similarity to susceptible strains when using different numbers of reference sequences. (A), Spearman’s ρ between epitope similarity and Ab neutralization breadth. The epitope similarity is estimated based on sequences in the 136 panel, from which the most susceptible strains were selected and based on which the similarity threshold was set. Row labels indicate weights. Columns indicate the number of susceptible reference strains tested with strains selected as those with the lowest IC50 values in neutralization assays. (B), the epitope similarity is estimated for group M sequences, using the three most susceptible strains from the 136 panel and the similarity threshold determined based on the panel of 136 strains tested in neutralization assays.
(TIFF)
