# Supplementary material for: The breadth of HIV-1 neutralizing antibodies depends on the conservation of key sites in their epitopes
Source: PLoS Comput Biol. 2019 Jun 6;15(6):e1007056. doi: 10.1371/journal.pcbi.1007056 (PMC6581281; doi:10.1371/journal.pcbi.1007056)
Supplement: S1 Table — The targets of the Abs on Env, their neutralization breadth as measured by Doria-Rose and colleagues [21] and the names of the Ab:Env complexes in the PDB archive are listed. (DOCX) [file pcbi.1007056.s001.docx]

**S1 Table. Thirty-four Abs included in the study.** The targets of the Abs on Env, their neutralization breadth as measured by Doria-Rose and colleagues [1] and the names of the Ab:Env complexes in the PDB archive are listed.

|  | Class | Breadth(%) | Complexes [pdb(Ag:Ab)] |
| --- | --- | --- | --- |
| HJ16 | CD4 binding site | 33.8 | 4ye4(G:HL) |
| b12 | CD4 binding site | 39.0 | 5vn8(G:HL), 5vn8(D:FJ), 5vn8(E:KI), 2ny7(G:HL) |
| VRC06b | CD4 binding site | 40.4 | 4xnz(G:HL), 4xnz(A:BC), 4xnz(D:EF) |
| 1B2530 | CD4 binding site | 42.6 | 4yfl(G:HL), 4yfl(E:FI) |
| CH103 | CD4 binding site | 44.1 | 4jan(G:HL), 4jan(I:BA) |
| 8ANC134 | CD4 binding site | 47.8 | 4rx4(G:HL), 4rx4(E:AD) |
| VRC03 | CD4 binding site | 47.8 | 3se8(G:HL) |
| VRC16 | CD4 binding site | 55.9 | 4ydk(G:HL) |
| VRC23 | CD4 binding site | 62.5 | 4j6r(G:HL) |
| 8ANC131 | CD4 binding site | 72.1 | 4rwy(A:HL) |
| VRC-PG20 | CD4 binding site | 75.0 | 4lsu(G:HL) |
| VRC-PG04 | CD4 binding site | 79.0 | 3se9(G:HL), 4i3r(G:HL), 4i3s(G:HL) |
| VRC-CH31 | CD4 binding site | 80.9 | 4lsp(G:HL), 4lsq(G:HL) |
| VRC27 | CD4 binding site | 81.6 | 4ydi(G:HL) |
| 3BNC117 | CD4 binding site | 82.4 | 4jpv(G:HL), 4lsv(G:HL), 5v8l(AC:HL), 5v8l(AD:IM), 5v8l(CD:GK), 5v8m(FG:RT), 5v8m(AG:US), 5v8m(AF:HL) |
| VRC18 | CD4 binding site | 82.4 | 4ydl(G:HL), 4ydl(A:BC) |
| VRC13 | CD4 binding site | 83.1 | 4ydj(I:AB), 4ydj(G:HL) |
| NIH45-46 | CD4 binding site | 86.8 | 3u7y(G:HL), 5d9q(G:D), 5d9q(J:O), 5d9q(A:I) |
| 12A21 | CD4 binding site | 87.5 | 4jpw(G:HL) |
| VRC01 | CD4 binding site | 91.4 | 3ngb(A:BC), 3ngb(G:HL), 3ngb(D:EF), 3ngb(I:JK), 4lst(G:HL), 5fyj(FG:UV), 5fyk(FG:UV) |
| VRC07 | CD4 binding site | 94.6 | 4olu(G:HL) |
| 2F5 | MPER | 52.2 | 1tji(P:HL), 1tjg(P:HL), 1tjh(P:HL) |
| 4E10 | MPER | 97.1 | 4xbe(P:HL), 4xaw(P:HL), 4wy7(P:HL), 2fx7(P:HL) |
| 10E8 | MPER | 97.4 | 4g6f(F:BD), 4g6f(P:HL), 5ghw(P:HL) |
| CH04 | V1V2-glycan | 44.1 | 5esz(G:HL), 5esz(C:AB) |
| PGT145 | V1V2-glycan | 73.5 | 5v8l(ACD:NJ) |
| PG16 | V1V2-glycan | 74.3 | 4dqo(C:HL) |
| PG9 | V1V2-glycan | 77.6 | 3u2s(G:HL), 3u2s(C:AB), 3u4e(G:HL), 3u4e(J:AB) |
| PGT135 | V3-glycan | 31.6 | 4jm2(E:AB) |
| PGT128 | V3-glycan | 61.8 | 5aco(A:HL), 5aco(D:IK), 5aco(C:GJ), 5c7k(C:AF), 5js9(C:AB), 5jsa(C:AB) |
| 10-1074 | V3-glycan | 65.4 | 5t3x(G:HL), 5t3z(G:HL) |
| 35O22 | gp120-gp41 interface | 39.0 | 4tvp(GB:DE), 5fyj(GB:DE), 5fyl(GB:DE), 5fyk(GB:DE), 5cez(GB:DE), 5t3s(BG:DE), 5utf(GB:DE), 5uty(GB:DE), 5um8(GB:DE), 5u7m(GB:DE), 5u7o(GB:DE), 5v7j(GB:DE), 5w6d(GB:DE) |
| 8ANC195 | gp120-gp41 interface | 59.6 | 4p9h(G:HL), 5c7k(CD:EB), 5cjx(KJ:AC), 5cjx(GB:HL), 5cjx(YX:DE), 5js9(CD:EF), 5jsa(CD:EF) |
| PGT151 | gp120-gp41 interface | 68.4 | 5fuu(FCD:HL), 5fuu(BFE:NM) |

1. Doria-Rose NA, Altae-Tran HR, Roark RS, Schmidt SD, Sutton MS, Louder MK, et al. Mapping Polyclonal HIV-1 Antibody Responses via Next-Generation Neutralization Fingerprinting. PLoS Pathog. 2017;13(1):e1006148. doi: 10.1371/journal.ppat.1006148. PubMed PMID: 28052137; PubMed Central PMCID: PMCPMC5241146.
