# Supplementary material for: The breadth of HIV-1 neutralizing antibodies depends on the conservation of key sites in their epitopes
Source: PLoS Comput Biol. 2019 Jun 6;15(6):e1007056. doi: 10.1371/journal.pcbi.1007056 (PMC6581281; doi:10.1371/journal.pcbi.1007056)
Supplement: S2 Table — Spearman’s ρ and adjusted p-values (in parentheses) are presented. Holm–Bonferroni method was used for multiple test adjustment within each sequence dataset. (DOCX) [file pcbi.1007056.s002.docx]

**S2 Table. Relationship between neutralization breadth and different Env epitope diversities.** Spearman’s ρ and adjusted p-values (in parentheses) are presented. Holm–Bonferroni method was used for multiple test adjustment within each sequence dataset.

|  | 136panel | gp M | A1 | B | C | D | 01_AE |
| --- | --- | --- | --- | --- | --- | --- | --- |
| no_weight | -0.12 (0.853) | -0.20 (0.701) | -0.11 (0.993) | -0.16 (1.000) | -0.10 (1.000) | -0.31 (0.167) | -0.08 (1.000) |
| no_weight.norm | -0.43 (0.078) | -0.44 (0.079) | -0.36 (0.285) | -0.38 (0.216) | -0.37 (0.322) | -0.38 (0.140) | -0.31 (0.688) |
| w.natoms | -0.28 (0.436) | -0.27 (0.549) | -0.24 (0.699) | -0.18 (1.000) | -0.21 (1.000) | -0.40 (0.140) | -0.15 (1.000) |
| w.npairs | -0.33 (0.345) | -0.31 (0.448) | -0.29 (0.554) | -0.23 (0.926) | -0.24 (1.000) | -0.41 (0.140) | -0.20 (1.000) |
| w.asa | -0.14 (0.853) | -0.17 (0.701) | -0.12 (0.993) | -0.12 (1.000) | -0.07 (1.000) | -0.35 (0.167) | -0.08 (1.000) |
| w.nnbs | -0.21 (0.694) | -0.21 (0.701) | -0.19 (0.873) | -0.15 (1.000) | -0.13 (1.000) | -0.34 (0.167) | -0.16 (1.000) |
| w.natoms.norm | -0.50 (0.034) | -0.49 (0.037) | -0.40 (0.231) | -0.41 (0.156) | -0.38 (0.315) | -0.50 (0.029) | -0.35 (0.527) |
| w.npairs.norm | -0.51 (0.030) | -0.48 (0.038) | -0.42 (0.161) | -0.43 (0.124) | -0.39 (0.272) | -0.50 (0.028) | -0.35 (0.527) |
| w.asa.norm | -0.45 (0.064) | -0.49 (0.036) | -0.36 (0.285) | -0.44 (0.110) | -0.36 (0.351) | -0.51 (0.023) | -0.28 (0.739) |
| w.nnbs.norm | -0.49 (0.038) | -0.54 (0.013) | -0.40 (0.231) | -0.52 (0.021) | -0.45 (0.107) | -0.54 (0.013) | -0.36 (0.461) |
| top9.natoms | -0.47 (0.048) | -0.46 (0.056) | -0.39 (0.231) | -0.38 (0.216) | -0.34 (0.369) | -0.40 (0.140) | -0.29 (0.726) |
| top9.npairs | -0.46 (0.057) | -0.42 (0.087) | -0.39 (0.231) | -0.39 (0.214) | -0.34 (0.369) | -0.43 (0.104) | -0.35 (0.527) |
| top9.asa | -0.30 (0.436) | -0.28 (0.549) | -0.27 (0.585) | -0.34 (0.303) | -0.18 (1.000) | -0.33 (0.167) | -0.19 (1.000) |
| top9.nnbs | -0.73 (1.2e-5) | -0.70 (5.0e-5) | -0.65 (3.8e-4) | -0.70 (4.9e-5) | -0.57 (6.1e-3) | -0.65 (4.0e-4) | -0.61 (1.6e-3) |
