# Supplementary material for: The breadth of HIV-1 neutralizing antibodies depends on the conservation of key sites in their epitopes
Source: PLoS Comput Biol. 2019 Jun 6;15(6):e1007056. doi: 10.1371/journal.pcbi.1007056 (PMC6581281; doi:10.1371/journal.pcbi.1007056)
Supplement: S3 Table — Spearman’s ρ and adjusted p-values (in parentheses) are presented. Holm–Bonferroni method was used for multiple test adjustment within each sequence dataset. CD4bs and non-CD4bs antibodies are shown separately. (DOCX) [file pcbi.1007056.s003.docx]

**S3 Table. Relationship between neutralization breadth and different Env epitope diversities.** Spearman’s ρ and adjusted p-values (in parentheses) are presented. Holm–Bonferroni method was used for multiple test adjustment within each sequence dataset. CD4bs and non-CD4bs antibodies are shown separately.

|  |  | 136panel | gp M | A1 | B | C | D | 01_AE |
| --- | --- | --- | --- | --- | --- | --- | --- | --- |
| CD4bs antibodies | no_weight | -0.04 (0.87) | -0.10 (0.67) | -0.06 (0.79) | -0.14 (0.53) | -0.14 (0.54) | -0.11 (0.64) | -0.11 (0.63) |
|  | no_weight.norm | -0.20 (0.39) | -0.25 (0.28) | -0.17 (0.45) | -0.17 (0.45) | -0.27 (0.24) | -0.12 (0.61) | -0.19 (0.40) |
|  | w.natoms | -0.37 (0.10) | -0.34 (0.13) | -0.26 (0.26) | -0.33 (0.15) | -0.36 (0.11) | -0.24 (0.29) | -0.26 (0.26) |
|  | w.npairs | -0.40 (0.07) | -0.42 (0.06) | -0.38 (0.09) | -0.33 (0.14) | -0.41 (0.07) | -0.32 (0.16) | -0.34 (0.13) |
|  | w.asa | -0.22 (0.34) | -0.13 (0.58) | -0.15 (0.53) | -0.10 (0.65) | -0.10 (0.66) | -0.21 (0.37) | -0.15 (0.51) |
|  | w.nnbs | -0.31 (0.18) | -0.30 (0.19) | -0.28 (0.23) | -0.24 (0.30) | -0.24 (0.30) | -0.30 (0.19) | -0.27 (0.23) |
|  | w.natoms.norm | -0.37 (0.10) | -0.37 (0.10) | -0.26 (0.25) | -0.25 (0.27) | -0.27 (0.23) | -0.26 (0.26) | -0.24 (0.30) |
|  | w.npairs.norm | -0.38 (0.09) | -0.33 (0.15) | -0.30 (0.19) | -0.24 (0.29) | -0.27 (0.24) | -0.27 (0.25) | -0.26 (0.25) |
|  | w.asa.norm | -0.32 (0.15) | -0.41 (0.07) | -0.22 (0.33) | -0.29 (0.20) | -0.31 (0.17) | -0.33 (0.14) | -0.27 (0.24) |
|  | w.nnbs.norm | -0.40 (0.07) | -0.47 (0.03) | -0.31 (0.18) | -0.49 (0.02) | -0.47 (0.03) | -0.41 (0.06) | -0.43 (0.05) |
|  | top9.natoms | -0.36 (0.10) | -0.38 (0.09) | -0.26 (0.26) | -0.22 (0.33) | -0.28 (0.21) | -0.22 (0.33) | -0.22 (0.35) |
|  | top9.npairs | -0.35 (0.12) | -0.29 (0.20) | -0.28 (0.23) | -0.21 (0.37) | -0.25 (0.28) | -0.26 (0.25) | -0.26 (0.26) |
|  | top9.asa | -0.09 (0.69) | -0.14 (0.54) | -0.16 (0.48) | -0.05 (0.83) | -0.07 (0.76) | -0.13 (0.57) | -0.12 (0.60) |
|  | top9.nnbs | -0.73 (1.6e-4) | -0.73 (1.6e-4) | -0.68 (7.7e-4) | -0.71 (3.1e-4) | -0.61 (3.3e-3) | -0.61 (3.1e-3) | -0.74 (1.1e-4) |
| non-CD4bs antibodies | no_weight | -0.66 (0.18) | -0.61 (0.35) | -0.49 (0.90) | -0.52 (0.92) | -0.61 (0.38) | -0.53 (0.33) | -0.50 (1.00) |
|  | no_weight.norm | -0.64 (0.21) | -0.64 (0.27) | -0.45 (1.00) | -0.42 (1.00) | -0.38 (1.00) | -0.58 (0.33) | -0.35 (1.00) |
|  | w.natoms | -0.25 (0.83) | -0.23 (1.00) | -0.30 (1.00) | -0.07 (1.00) | -0.14 (1.00) | -0.57 (0.33) | -0.14 (1.00) |
|  | w.npairs | -0.24 (0.83) | -0.16 (1.00) | -0.19 (1.00) | -0.02 (1.00) | -0.14 (1.00) | -0.41 (0.33) | -0.19 (1.00) |
|  | w.asa | -0.53 (0.49) | -0.58 (0.47) | -0.40 (1.00) | -0.45 (1.00) | -0.54 (0.75) | -0.63 (0.27) | -0.41 (1.00) |
|  | w.nnbs | -0.52 (0.49) | -0.50 (0.61) | -0.32 (1.00) | -0.32 (1.00) | -0.45 (1.00) | -0.60 (0.32) | -0.38 (1.00) |
|  | w.natoms.norm | -0.48 (0.49) | -0.36 (1.00) | -0.43 (1.00) | -0.33 (1.00) | -0.31 (1.00) | -0.51 (0.33) | -0.30 (1.00) |
|  | w.npairs.norm | -0.41 (0.49) | -0.34 (1.00) | -0.39 (1.00) | -0.27 (1.00) | -0.34 (1.00) | -0.47 (0.33) | -0.27 (1.00) |
|  | w.asa.norm | -0.51 (0.49) | -0.52 (0.61) | -0.45 (1.00) | -0.43 (1.00) | -0.35 (1.00) | -0.58 (0.33) | -0.35 (1.00) |
|  | w.nnbs.norm | -0.47 (0.49) | -0.45 (0.73) | -0.41 (1.00) | -0.37 (1.00) | -0.36 (1.00) | -0.57 (0.33) | -0.35 (1.00) |
|  | top9.natoms | -0.59 (0.34) | -0.37 (1.00) | -0.54 (0.77) | -0.46 (1.00) | -0.26 (1.00) | -0.59 (0.33) | -0.33 (1.00) |
|  | top9.npairs | -0.60 (0.34) | -0.53 (0.61) | -0.50 (0.90) | -0.43 (1.00) | -0.37 (1.00) | -0.56 (0.33) | -0.40 (1.00) |
|  | top9.asa | -0.60 (0.34) | -0.51 (0.61) | -0.53 (0.77) | -0.53 (0.85) | -0.31 (1.00) | -0.62 (0.30) | -0.38 (1.00) |
|  | top9.nnbs | -0.66 (0.18) | -0.56 (0.51) | -0.54 (0.77) | -0.48 (1.00) | -0.39 (1.00) | -0.64 (0.25) | -0.40 (1.00) |
