# Supplementary material for: The breadth of HIV-1 neutralizing antibodies depends on the conservation of key sites in their epitopes
Source: PLoS Comput Biol. 2019 Jun 6;15(6):e1007056. doi: 10.1371/journal.pcbi.1007056 (PMC6581281; doi:10.1371/journal.pcbi.1007056)
Supplement: S4 Table — The epitope similarity is estimated based on sequences in the 136 panel. Spearman’s ρ and adjusted p-values (in parentheses) are presented. Holm–Bonferroni method was used for multiple test adjustment. (DOCX) [file pcbi.1007056.s004.docx]

**S4 Table. Relationship between neutralization breadth and percentage of similar epitope to susceptible strains.** The epitope similarity is estimated based on sequences in the 136 panel. Spearman’s ρ and adjusted p-values (in parentheses) are presented. Holm–Bonferroni method was used for multiple test adjustment.

|  | All Abs | CD4bs Abs | non-CD4bs Abs |
| --- | --- | --- | --- |
| no_weight | 0.60 (1.9e-3) | 0.52 (0.122) | 0.58 (0.390) |
| no_weight.norm | 0.31 (0.071) | 0.32 (0.317) | 0.39 (0.935) |
| w.natoms | 0.44 (0.035) | 0.42 (0.169) | 0.26 (1.000) |
| w.npairs | 0.51 (0.016) | 0.59 (0.044) | 0.24 (1.000) |
| w.asa | 0.56 (5.4e-3) | 0.50 (0.128) | 0.57 (0.390) |
| w.nnbs | 0.61 (1.6e-3) | 0.61 (0.042) | 0.46 (0.899) |
| w.natoms.norm | 0.44 (0.035) | 0.49 (0.128) | 0.39 (0.935) |
| w.npairs.norm | 0.48 (0.021) | 0.52 (0.122) | 0.25 (1.000) |
| w.asa.norm | 0.50 (0.016) | 0.47 (0.128) | 0.46 (0.899) |
| w.nnbs.norm | 0.51 (0.016) | 0.62 (0.036) | 0.45 (0.899) |
| top9.natoms | 0.64 (4.6e-4) | 0.59 (0.044) | 0.69 (0.114) |
| top9.npairs | 0.67 (1.7e-4) | 0.60 (0.043) | 0.70 (0.104) |
| top9.asa | 0.43 (0.035) | 0.28 (0.317) | 0.68 (0.114) |
| top9.nnbs | 0.80 (2.7e-7) | 0.78 (5.2e-4) | 0.73 (0.070) |
