# Supplementary figures and images for: The breadth of HIV-1 neutralizing antibodies depends on the conservation of key sites in their epitopes
Source: PLoS Comput Biol. 2019 Jun 6;15(6):e1007056. doi: 10.1371/journal.pcbi.1007056 (PMC6581281; doi:10.1371/journal.pcbi.1007056)

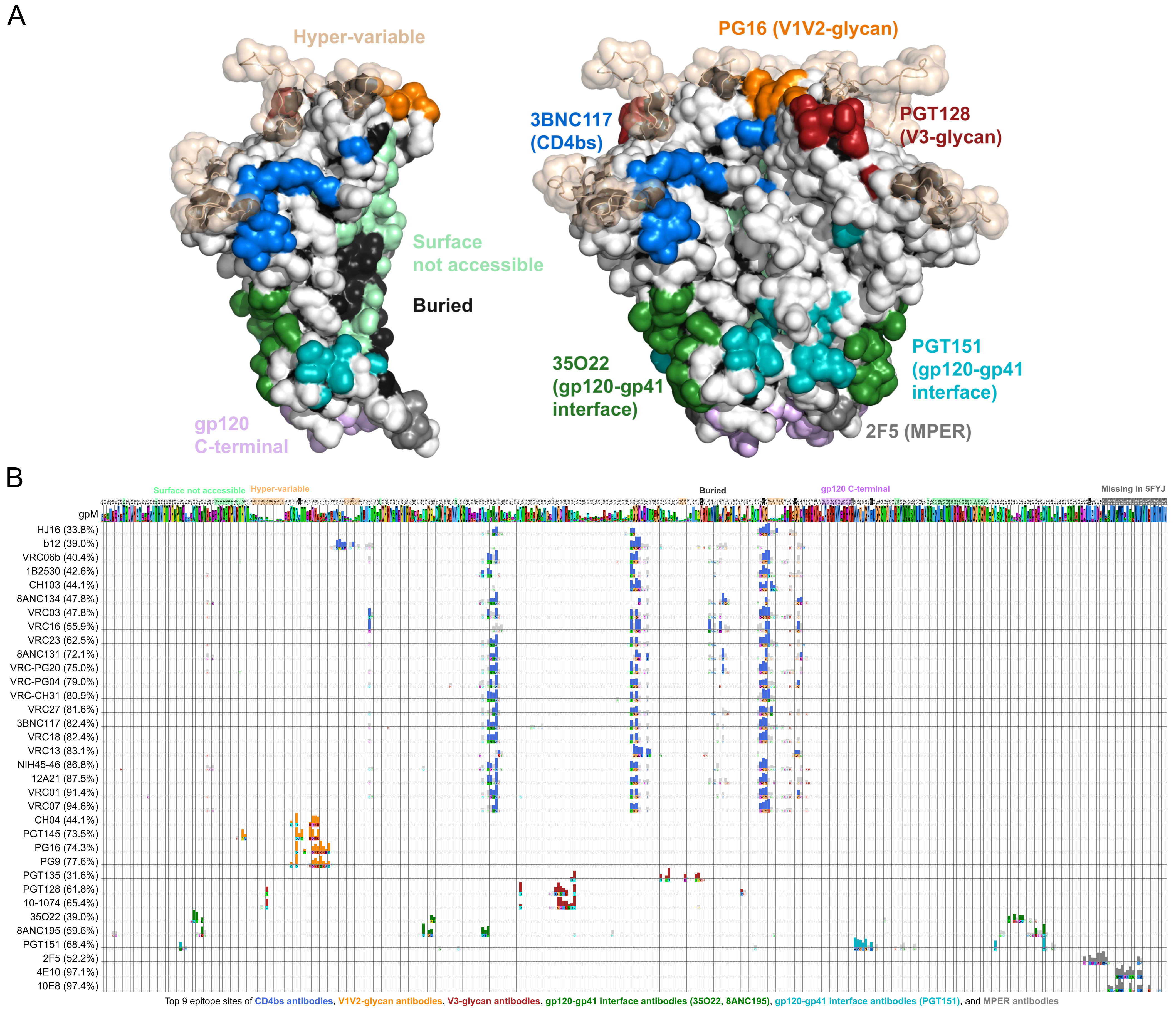

Supplement: S1 Fig — (A) Representative bnAb epitopes from our dataset are represented on the structure 5FYJ. (B) Epitope sites on the Env surface sites. The name and breadth of the 34 Abs analyzed are figured as row headers. Histograms correspond to the number of neighbor antibody residues, with the top nine sites colored by epitope categories. Buried sites are included only if they are among the top nine sites. 5FYJ was used for the structure rendering and the surface detection. (TIFF) [file pcbi.1007056.s005.tiff]

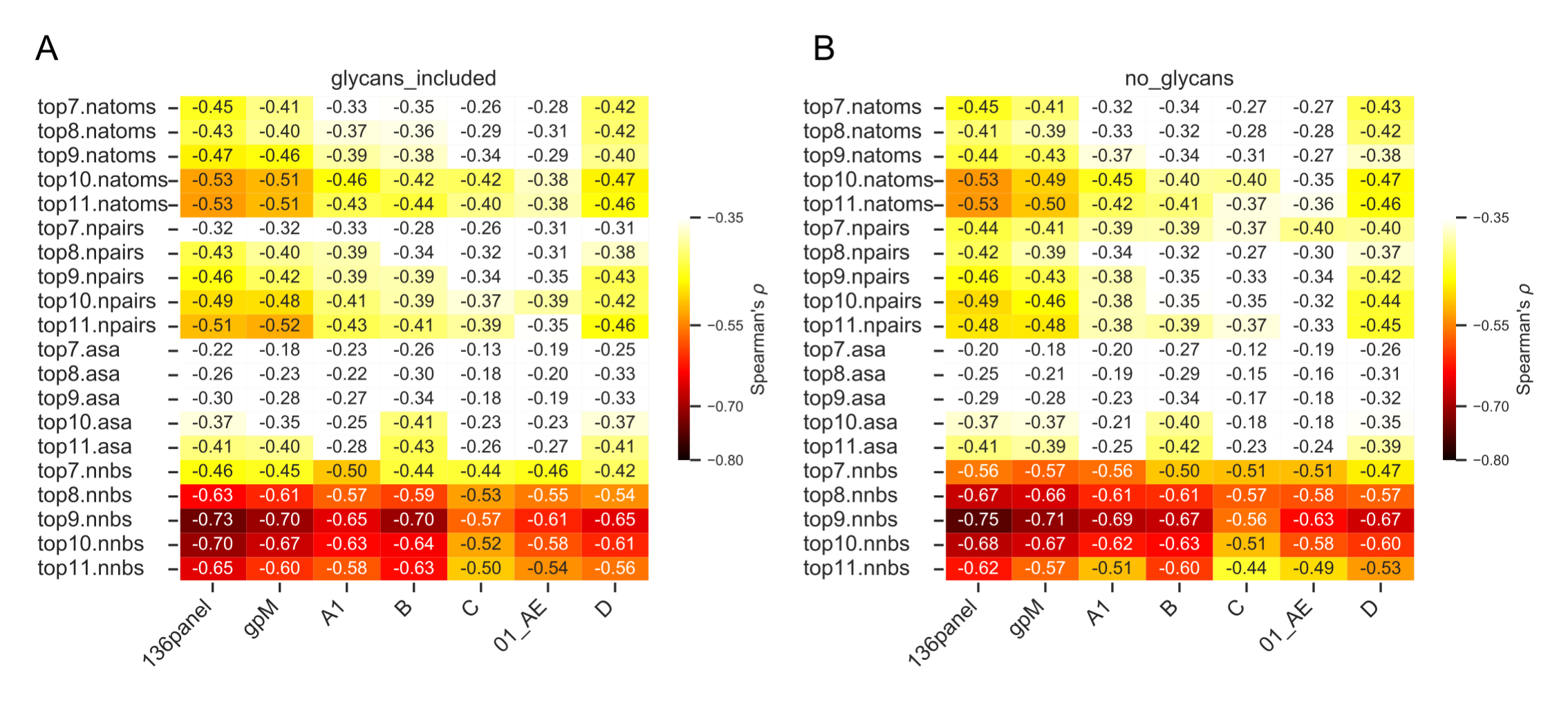

Supplement: S2 Fig — Epitope diversity values were estimated using different Env dataset (columns) and different sets of top sites (rows). Spearman’s ρ coefficients corresponding to the relationship between neutralization breadth and Env epitope diversity are presented with (A) and without glycans (B). Each cell is color-coded for ρ values with a p-value < 0.05. Epitope diversity values of the top nine sites as ranked by the number of neighbor antibody residues showed the highest correlation with the Ab neutralization breadth. (TIFF) [file pcbi.1007056.s006.tiff]

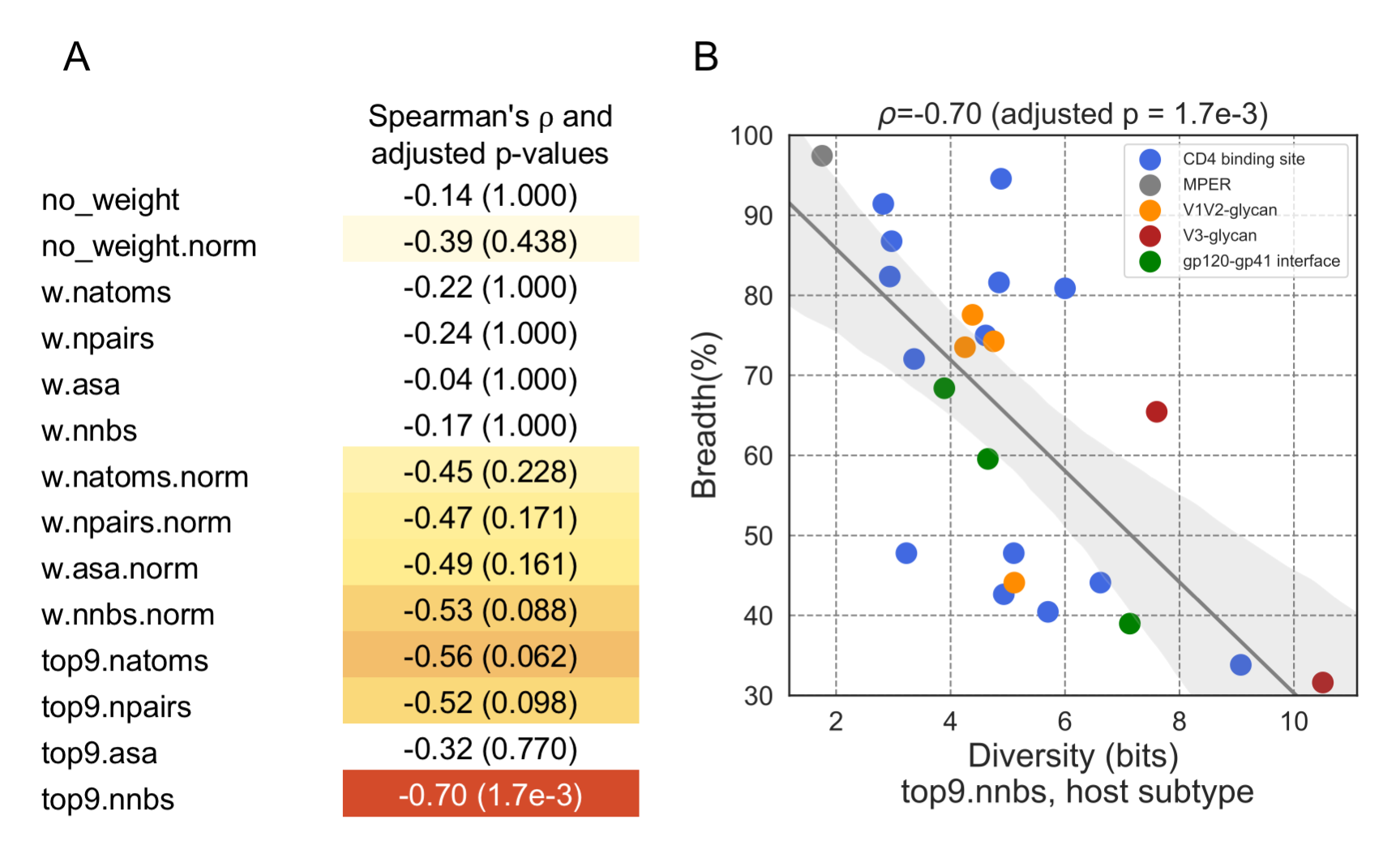

Supplement: S3 Fig — (A), relationship between neutralization breadth and the epitope diversity that is estimated from the host subtype sequences using different weighting schemes (rows). Spearman’s ρ and adjusted p-values (Holm–Bonferroni method, in parenthesis) are presented. (B), the neutralization breadth versus epitope diversity of top nine epitope sites estimated from the host subtype sequences. The 95% confidence interval of the linear regression line was determined by 1000 bootstrap replicates. (TIFF) [file pcbi.1007056.s007.tiff]

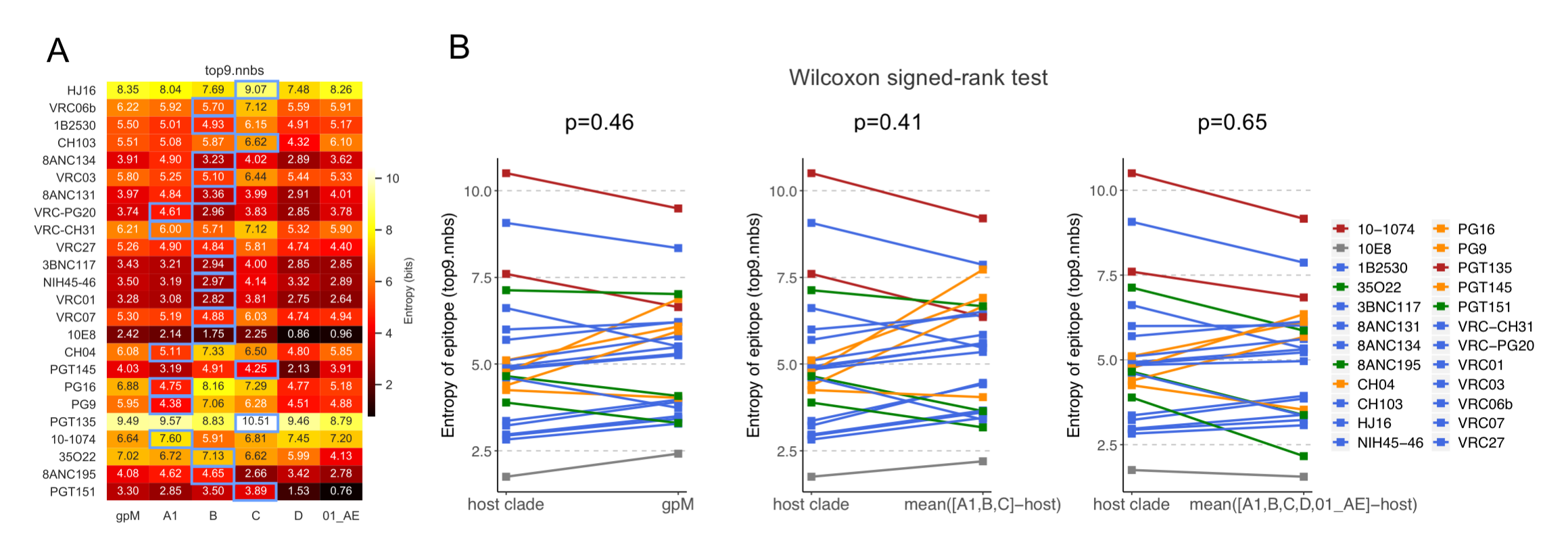

Supplement: S4 Fig — (A), the diversity of the top nine epitope sites (ranked by number of neighbor antibody residues) is shown as a heatmap with antibodies in rows and Env alignments in columns. The host subtype is indicated by blue rectangles in the heatmap. (B), the three panels on the right compare the epitope diversity of the host subtype against other subtypes (with corresponding host subtypes excluded). (TIFF) [file pcbi.1007056.s008.tiff]

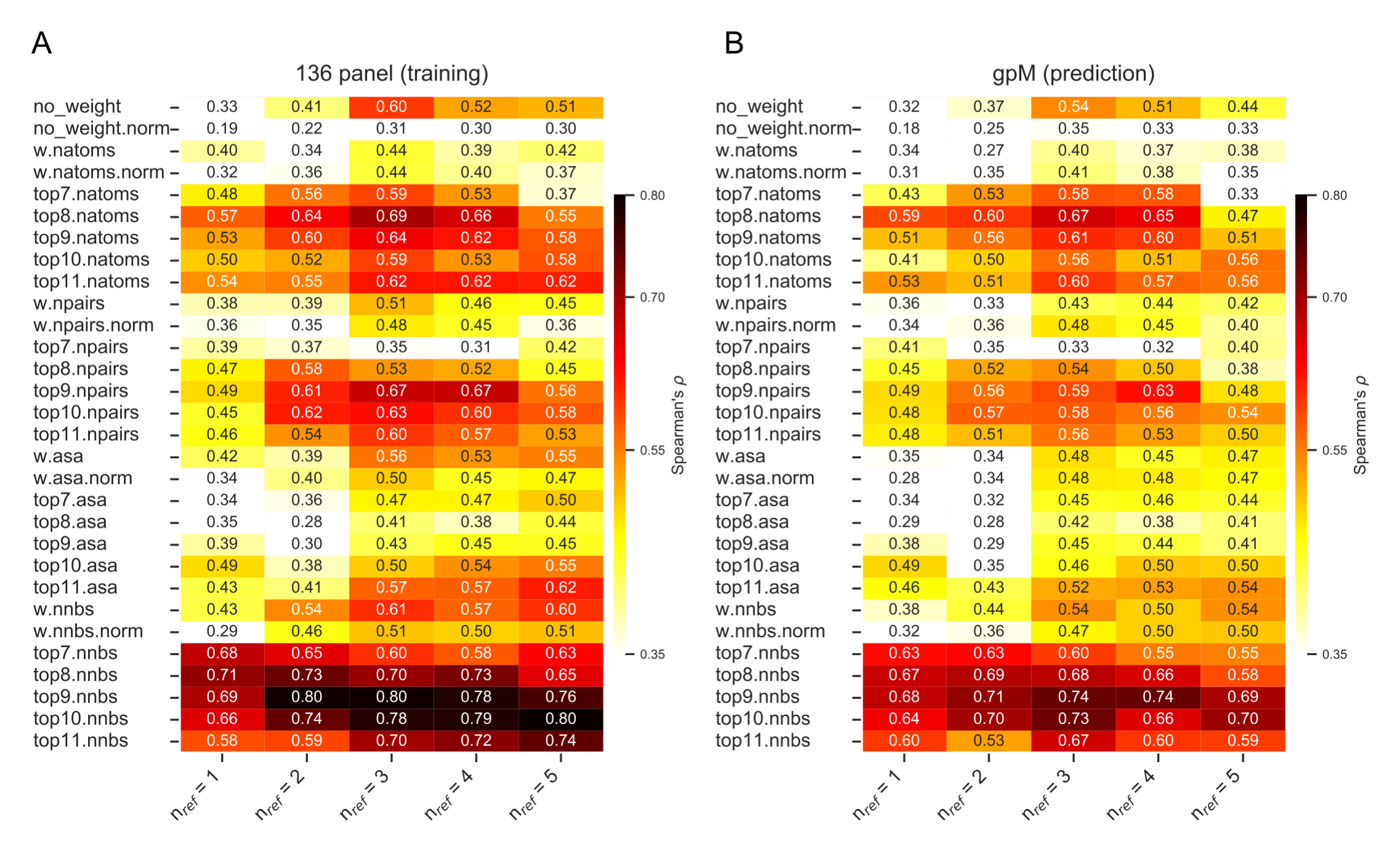

Supplement: S5 Fig — (A), Spearman’s ρ between epitope similarity and Ab neutralization breadth. The epitope similarity is estimated based on sequences in the 136 panel, from which the most susceptible strains were selected and based on which the similarity threshold was set. Row labels indicate weights. Columns indicate the number of susceptible reference strains tested with strains selected as those with the lowest IC50 values in neutralization assays. (B), the epitope similarity is estimated for group M sequences, using the three most susceptible strains from the 136 panel and the similarity threshold determined based on the panel of 136 strains tested in neutralization assays. (TIFF) [file pcbi.1007056.s009.tiff]
